# Impact of corticosteroid administration on glioblastoma progression before and after adjuvant treatments: recent updates on contradictory findings and mechanistic interactions

**DOI:** 10.3389/fonc.2026.1774736

**Published:** 2026-03-02

**Authors:** Maher Kurdi, Ali Kabli, Alaa Alkhotani, Amal Alkhotani, Rakan Bokhari, Zayd Jastaniah, Razan Amjad, Huda Althoukhi, Taghreed Alsinani, Hussain Alamoudi, Saleh Baeesa

**Affiliations:** 1 Department of Pathology, Faculty of Medicine, King Abdulaziz University, Rabigh, Saudi Arabia; 2 Department of Physiology, Faculty of Medicine, King Abdulaziz University, Rabigh, Saudi Arabia; 3 Department Pathology, College of Medicine, Umm Al-Qura University, Makkah, Saudi Arabia; 4 Department of Medicine, College of Medicine, Umm Al-Qura University, Makkah, Saudi Arabia; 5 Department of Surgery, Faculty of Medicine, King Abdulaziz University, Jeddah, Saudi Arabia; 6 Department of Internal Medicine, Faculty of Medicine, King Abdulaziz University, Rabigh, Saudi Arabia; 7 Department of Internal Medicine, Faculty of Medicine, King Abdulaziz University, Jeddah, Saudi Arabia; 8 Department of Neurosurgery, King Fahad General Hospital, Jeddah, Saudi Arabia; 9 Oncology Center, East Jeddah Hospital, Jeddah, Saudi Arabia; 10 Department of Neurosciences, King Faisal Specialist Hospital and Research Center, Jeddah, Saudi Arabia

**Keywords:** corticosteroids, dexamethasone, glioblastoma, grade 4 astrocytoma, progression, temozolomide resistance

## Abstract

Corticosteroids, particularly dexamethasone (DEX), are widely used in the supportive management of glioblastoma and grade 4 astrocytoma because of their rapid efficacy in reducing vasogenic cerebral edema and alleviating neurological symptoms. Despite these benefits, their impact on tumor biology and treatment response remains highly controversial. While experimental studies have reported anti-proliferative and anti-migratory effects of DEX in glioma models, accumulating clinical and translational evidence suggests detrimental interactions with radiotherapy (RT) and temozolomide (TMZ), particularly when steroids are administered at higher doses or during RT. Proposed mechanisms include induction of chemoresistance, suppression of antitumor immune responses, and modulation of DNA damage repair pathways within the tumor microenvironment. Recent data implicate steroid receptor coactivator-1 (SRC-1) as a key molecular mediator linking corticosteroid signaling to immune regulation and tumor recurrence, highlighting a novel microenvironmental mechanism independent of steroid dose. Emerging therapeutic strategies, including agents targeting epigenetic regulators, metabolic pathways, or repurposed drugs such as Riluzole, Metformin, Mifepristone, and Chlorpromazine, show promise in mitigating steroid-associated resistance to TMZ. Collectively, these findings emphasize the complex, context-dependent role of corticosteroids in glioblastoma or grade 4 astrocytoma and emphasize the need for optimized dosing, timing, and integrated treatment strategies to improve patient outcomes.

## Introduction

The management of patients with grade 4 astrocytoma or glioblastoma remains challenging; however, contemporary multimodal treatment strategies have led to improved outcomes. Median overall survival now approaches approximately 30 months, with a reported 5-year survival probability of about 25% (1). Survival is strongly influenced by extent of resection and molecular factors, particularly MGMT promoter methylation and the absence of TERT promoter mutations (2). Before 2016, tumor diagnosis relied predominantly on histopathological assessment supported by immunohistochemistry. Core histological features included increased mitotic activity, microvascular proliferation, and geographic necrosis (3). The identification of isocitrate dehydrogenase (*IDH*) mutations subsequently enabled the classification of glioblastomas into IDH-mutant and IDH-wildtype categories. In the 2021 World Health Organization (WHO) Classification of Tumors of the Central Nervous System (5th edition), IDH-mutant astrocytoma, grade 4, was formally separated from IDH-wildtype glioblastoma (3). The integration of molecular diagnostics, including IDH and alpha-thalassemia/mental retardation syndrome X-linked (*ATRX*) status, has facilitated the stratification of diffuse malignant astrocytomas into biologically distinct subgroups. Glioblastoma is now recognized as a separate entity, distinct from grade 4 astrocytoma, and is defined by a wild-type IDH status despite overlapping histomolecular characteristics (2, 3). According to the Consortium to Inform Molecular and Practical Approaches to CNS Tumor Taxonomy (cIMPACT-NOW), a definitive diagnosis of WHO grade 4 astrocytoma requires the presence of an IDH mutation, loss of ATRX expression, TP53 mutation, and the absence of 1p/19q codeletion. In contrast, glioblastomas are characterized by wild-type IDH and hallmark molecular alterations, including epidermal growth factor receptor (EGFR) amplification, telomerase reverse transcriptase (TERT) promoter mutations, and characteristic chromosomal changes, notably gain of chromosome 7 and loss of chromosome 10 (3, 4).

Prognosis is influenced by several factors, including patient age, preoperative performance status assessed by the Karnofsky Performance Scale, extent of surgical resection, and IDH mutational status (5–8). Gross total resection has been associated with better survival, while residual contrast-enhancing tumor on early postoperative Magnetic Resonance Imaging (MRI) predicts markedly reduced survival, conferring a six- to sevenfold increased risk of death (3, 7). Concurrent temozolomide (TMZ) with RT significantly improves overall and progression-free survival (PFS) in newly diagnosed glioblastoma or grade 4 astrocytoma, emphasizing that additional therapies combined with TMZ may either enhance treatment sensitivity or promote tumor progression (2, 9).

Beyond tumor-directed therapies, corticosteroids are routinely administered perioperatively and at terminal stages to alleviate tumor-associated cerebral edema (9). This vasogenic edema results from blood–brain barrier (BBB) breakdown, leading to plasma leakage, increased intracranial pressure (ICP), neurological deficits, and reduced chemotherapeutic penetration, particularly of TMZ (10, 11). However, the mechanistic interplay between steroid therapy—especially dexamethasone (DEX)—and TMZ efficacy in glioblastoma or grade 4 astrocytoma remains unclear. Conflicting evidence persists regarding the impact of steroids on patients’ survival and tumor recurrence, though recent studies have begun exploring steroid interactions within the glioma microenvironment and the potential modulatory effects of TMZ on steroid-related outcomes.

Corticosteroids, particularly DEX, are potent synthetic steroids widely used to manage vasogenic edema in patients with brain tumors (12). However, their impact on survival has not been extensively evaluated in randomized clinical trials (12, 13). Its use for cerebral edema was first introduced in 1952 by Ingraham and later by Kofman (14). Preoperative DEX administration typically alleviates edema-related symptoms within 48 hours (15). Although it is routinely prescribed preoperatively when radiological peritumoral edema causes significant neurological symptoms, it is sometime continued postoperatively or prior to RT in cases with marked mass effect (16). DEX is characterized by minimal mineralocorticoid activity, a long half-life, and high potency. Despite its widespread use, clear guidelines defining optimal dosing remain limited. For symptomatic patients, a maximum daily dose of 16 mg divided into four doses is recommended following surgery (17–19). Initial loading doses of 10–20 mg followed by maintenance dosing have also been proposed for acute presentations (20, 21). Careful monitoring for systemic adverse effects is essential, and tapering is advised once symptoms improve and before starting RT. Nevertheless, DEX use during RT remains controversial. Thus, dose balancing is suggested when daily intake reaches 16mg (21). In selected symptomatic glioblastoma patients or those with limited life expectancy, low-dose DEX (0.5–1.0 mg daily) may be continued after RT.

## Methodology

To ensure transparency and scientific rigor, we conducted a narrative review examining the impact of corticosteroid administration on glioblastoma progression before and after adjuvant treatments. A comprehensive literature search was performed using PubMed, Scopus, and Web of Science, covering studies published up to November 2025. The search strategy combined relevant keywords and Boolean operators (AND/OR), including “glioblastoma,” “grade 4 astrocytoma,” “corticosteroids,” “dexamethasone,” “radiotherapy,” “temozolomide,” “chemoresistance,” “tumor microenvironment,” and “immune modulation.”

Eligible publications included original experimental studies, clinical investigations, translational research, and narrative or systematic reviews published in English that addressed the biological or clinical effects of corticosteroids on glioblastoma progression, treatment response, or patient outcomes. Article selection was performed through initial screening of titles and abstracts, followed by full-text evaluation. Studies lacking relevance to corticosteroid–tumor interactions or adjuvant treatment effects were excluded.

Selected studies were analyzed qualitatively, with emphasis on molecular mechanisms, treatment timing and dosing, immune and metabolic modulation, and interactions with radiotherapy and temozolomide. Findings were synthesized into thematic sections addressing biological effects, clinical implications, resistance mechanisms, and emerging steroid-sparing or resistance-mitigating strategies. As a narrative review, no meta-analytic techniques were applied.

### Impact of corticosteroid on glioblastoma or grade 4 astrocytoma growth

The impact of corticosteroids on glioma cell growth and patient survival, whether administered before or after the initiation of chemoradiotherapy, remains highly controversial, with contradictory findings reported across studies. Experimental evidence is still limited and insufficient to draw definitive conclusions, largely because the complex biological interactions between steroid molecules delivered to the brain and glial tumor cells have not been fully elucidated. Nevertheless, corticosteroid therapy in glioblastoma provides pathological and radiological benefits beyond the reduction of peritumoral edema. Several studies have demonstrated tumor-suppressive effects of DEX in various glioma models, both *in vitro* and *in vivo* (22–24). The anti-proliferative properties of DEX have been documented across multiple glioma cell lines, including T98G, A172, 86HG39, F98, GL261, and U87. In these models, DEX inhibited cell proliferation in a dose-dependent manner and, in some cases, induced cell death (23). *In vivo* murine xenograft studies further showed that DEX treatment was associated with a reduction in tumor mass (25). Evidence regarding the effects of DEX on glioma cell migration and invasion remains similarly inconsistent and appears to be highly dependent on the specific cell type examined. DEX has been reported to suppress migration and invasion in several glioma cell lines, including C6, U251, U373, and A172 (26). In U87 cells, DEX reduced migratory capacity by decreasing matrix metalloproteinase-2 (MMP-2) secretion (27). Likewise, DEX inhibited migration and invasion of U373 cells through a glucocorticoid receptor–dependent ERK1/2 MAPK signaling pathway, a pathway known to play a central role in glioblastoma or grade 4 astrocytoma invasion, migration, and proliferation (24).

Unlike primary brain lymphoma, glioblastoma or grade 4 astrocytoma does not typically exhibit radiographic regression following systemic corticosteroid administration. Nevertheless, corticosteroid-induced regression of glioblastoma is a rare but well-documented radiological phenomenon, with few cases reported (28–32). Such steroid-related imaging changes create a diagnostic challenge, as they may delay biopsy due to suspicion of brain lymphoma. Cuoco et al. described a 76-year-old man who presented with a right parietal mass. Following DEX treatment, imaging revealed a marked reduction in lesion size and surrounding edema, closely mimicking brain lymphoma (33). Subsequent biopsy, however, confirmed glioblastoma, and the patient experienced rapid clinical deterioration thereafter. Similarly, Toda et al. reported a 71-year-old man with presumed glioblastoma who showed significant radiological regression after corticosteroid therapy, including reduced edema and contrast enhancement (34). After steroid withdrawal, lesion density and enhancement reappeared, illustrating corticosteroid-induced pseudoregression that complicated diagnosis and postponed definitive surgery. In a larger cohort, corticosteroid initiation was associated with a median reduction in contrast-enhancing tumor volume of 24% (35). After adjustment for scan intervals and comparison with controls, tumor shrinkage attributable to corticosteroids was estimated at approximately 44%, accompanied by a 14% reduction in apparent diffusion coefficient values. Collectively, these findings confirm that corticosteroid therapy can induce a significant radiological “pseudo response” in glioblastoma. Radiological pseudoregression in glioblastoma reflects treatment- or steroid-related transient reductions in enhancement and edema without true tumor control, whereas true regression represents genuine tumor cell loss with sustained radiologic improvement and corresponding clinical stability or response over time.

### Interaction between corticosteroid and radiotherapy or chemotherapy

Several studies have reported an unfavorable interaction between DEX and RT, or alkylating CT. Scattered evidence suggests that dependency on DEX during the post-surgical RT period may act as an independent negative prognostic factor in glioblastoma or grade 4 astrocytoma (36, 37). Corticosteroid use during RT has also been identified as an independent indicator of reduced overall survival (OS) (38). Watne et al. demonstrated that patients requiring corticosteroids after surgery and throughout RT experienced approximately a twofold increase in mortality compared with patients who did not require steroids after surgery (37). Other studies showed that patients who were not treated with DEX at the initiation of RT had a median survival of 20 months, whereas those receiving DEX had a reduced median survival of 13 months (38, 39). In addition, Wong et al. evaluated 119 patients with recurrent glioblastoma treated with tumor-treating alternating electric fields (TTFields) and found that DEX doses exceeding 4 mg/day were associated with shorter OS in both treatment groups (39). Collectively, these findings highlight that the interaction between steroid therapy, the tumor immune microenvironment, and radiation exposure remains incompletely understood, raising ongoing concerns regarding whether corticosteroid use during RT may compromise patient survival. Steroids administered at the initiation of radiotherapy may be associated with enhanced glioma cell survival and modulation of antitumor immune responses, potentially influencing tumor–microenvironment signaling. These effects could involve activation of glucocorticoid receptor–dependent pathways related to cell survival, DNA damage repair, and metabolic adaptation, which may partially counteract radiation-induced cytotoxic stress.

Moreover, several studies have shown that DEX can promote glioblastoma cell proliferation, induce a glioma stem cell–like phenotype, reduce host survival, and contribute to resistance against TMZ (16, 40). *In vitro* studies demonstrated that DEX protects glioblastoma cells from TMZ-induced apoptosis by inhibiting caspase cleavage and altering B-cell lymphoma-2 (Bcl-2) expression levels (41). In primary glioblastoma cell lines treated with TMZ, the addition of DEX antagonized TMZ-induced apoptosis and enhanced resistance to nutrient deprivation (42). Notably, the human O6-methylguanine-DNA methyltransferase (MGMT) promoter contains two glucocorticoid response elements, and recent evidence indicates that DEX-mediated induction of MGMT promoter activity represents a key mechanism underlying chemoresistance (43). A retrospective multivariate analysis of 73 glioblastoma patients identified concurrent DEX administration during radiochemotherapy as a significant risk factor for poor OS (44). High-dose DEX treatment has also been associated with transient radiological disappearance of tumor mass in some patients, followed by rapid tumor re-emergence with increased aggressiveness within 1–4 weeks (31). Shields et al. reported that DEX use during adjuvant radiochemotherapy correlated with reduced OS, except in patients receiving bevacizumab, where this adverse effect appeared attenuated (44). Consequently, discontinuation of DEX before initiating adjuvant TMZ has been recommended, although this decision remains clinician-dependent (44).

Importantly, none of these studies accounted for potential circadian variation in glucocorticoid activity or whether daily rhythmic activation of glucocorticoid signaling influences glioblastoma progression. Maria et al. demonstrated that glioblastoma or grade 4 astrocytoma growth is regulated by circadian rhythms (45). Glucocorticoid signaling exerts time-dependent effects on tumor progression. Disruption of circadian cues significantly slows tumor growth and improves outcomes (45). These findings raise critical questions regarding the timing of steroid administration relative to RT or TMZ exposure, as the impact of their concurrent use remains insufficiently elucidated in the current literature.

### Mechanistic reasoning and contradiction for the impact of DEX on CT or RT

The precise mechanisms through which corticosteroids reduce peritumoral edema or interact with TMZ and RT remain incompletely understood. In the tumor microenvironment, tumor-associated macrophages (TAMs) often encircle T cells, preventing effective interactions between tumor cells and CD8-positive T cells, thereby facilitating tumor growth and proliferation (46). After steroid administration, it is believed that steroid molecules reaching the brain tumor exert anti-inflammatory effects that suppress T-cell activity within the tumor microenvironment, potentially influencing TAM behavior (46). Steroid particles are therefore thought to directly contribute to T-cell inhibition (46). Whether DEX alters the efficacy of DNA-damaging therapies in glioblastoma also remains uncertain (47). During RT or CT, this interaction is thought to differ, as radiation beams or TMZ particles may interact with corticosteroid molecules through distinct biological mechanisms. Experimental studies have reported variable interactions between corticosteroids and CT, ranging from antagonistic to neutral or even synergistic effects (48). Investigations into the prognostic significance of T-cell infiltration in glioblastoma have produced conflicting findings. While several studies suggest that increased CD8^+^ cytotoxic T-cell accumulation may enhance tumor cell clearance, others have failed to confirm a survival benefit (49–51). Mauldin et al. identified CD8^+^ cytotoxic T-cells as independent predictors of OS; however, this relationship may be influenced by interactions between tumor-infiltrating lymphocytes (TILs) and TAMs (50). Kurdi et al. demonstrated that although TAMs can shield tumor cells and impairing cytotoxic T-cell recognition, thereby facilitating tumor evolution (52). The effect of steroid before or after starting the CT or RT was not clearly investigated. The regulatory mechanisms governing CD8^+^ T-cell infiltration remain poorly defined, and additional factors may contribute to cytotoxic T-cell downregulation. Although glucocorticoids are known to exert immunosuppressive effects, the direct relationship between corticosteroid therapy and T-cell regulation in glioblastoma or grade 4 astrocytoma requires more understanding (53, 54).

### The Relationship between SRC-1 and CD8 cytotoxic T-cells

**Figure 1:**
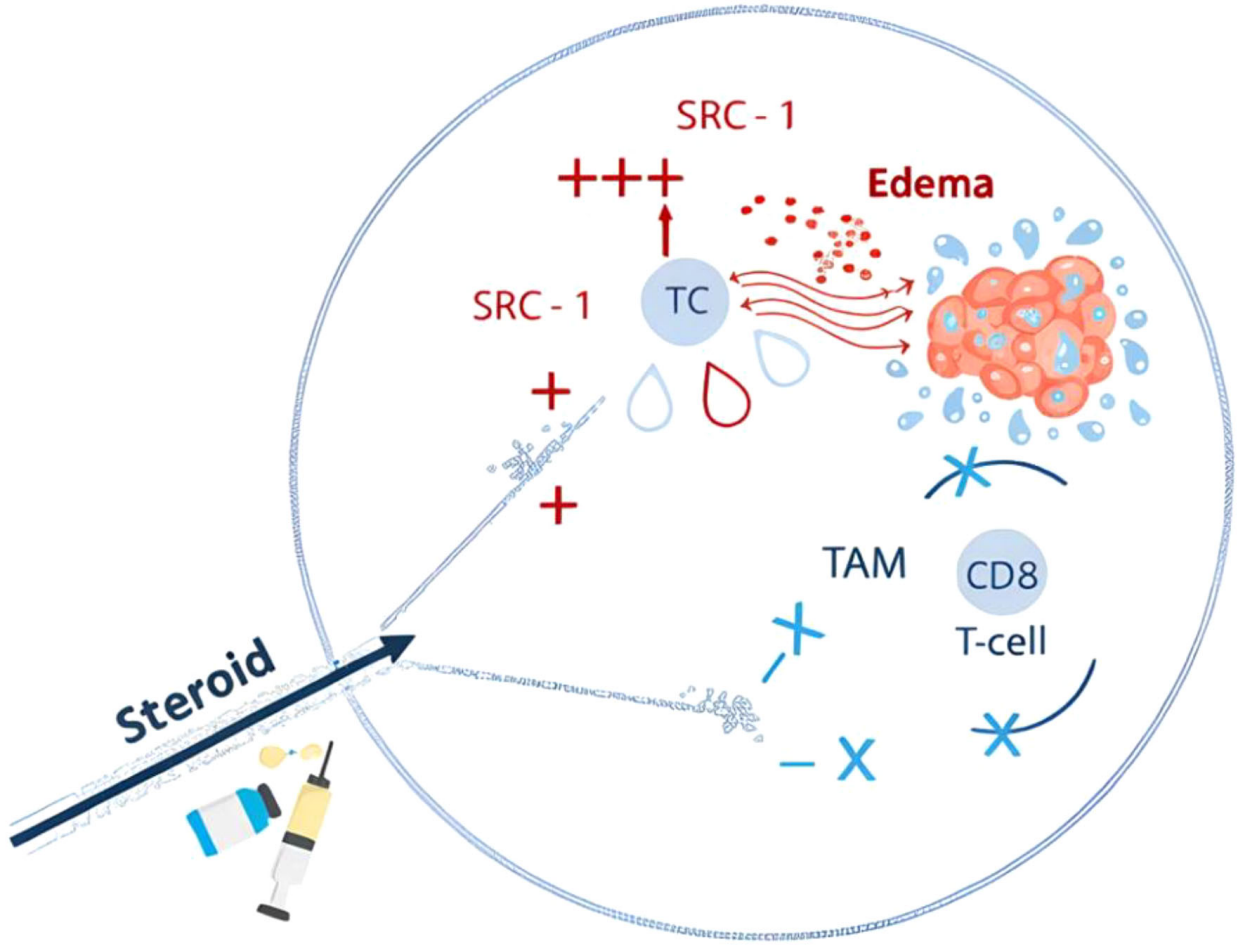
Proposed mechanistic interaction between corticosteroids, CD8^+^ T cells, TAMs, and SRC-1 in the glioblastoma tumor microenvironment. After reaching the tumor microenvironment, corticosteroids may not directly suppress CD8^+^ T cells because tumor-associated macrophages (TAMs) physically encircle and inhibit T-cell–tumor interactions. Steroids may instead bind SRC-1, altering tumor signaling, while TAM activation enhances immune suppression. Interactions with radiotherapy or temozolomide, including resistance mechanisms, remain poorly defined.

Steroid receptor coactivators (SRCs) represent one of the principal gene families implicated in steroid signaling. The p160 SRC family comprises SRC-1, SRC-2, and SRC-3 (55). SRC-1 was initially identified for its capacity to enhance transcriptional activity of nuclear receptors and regulates ligand-dependent transactivation of estrogen, androgen, and thyroid hormone receptors (56). Its function is tightly controlled by intracellular ubiquitination. SRC-1 is predominantly expressed in the thalamus and hippocampus and plays a physiological role in brain development (55, 56). Notably, SRC-1 expression is higher in the brain than SRC-2 or SRC-3 and is upregulated in several subcortical regions of the adult mouse brain (55, 57). Detection of SRC-1 in astrocytic tumors supports its expression in glial cells and suggests a potential association with grade 4 astrocytoma or glioblastoma biology, while SRC-2 and SRC-3 are also reported to be overexpressed in astrocytic neoplasms (56–58). In a cohort of 36 WHO grade 4 astrocytomas and glioblastomas, Kurdi et al. examined the effects of corticosteroids on the tumor microenvironment (59). In contrast to earlier reports, corticosteroid exposure was not associated with significant changes in CD8^+^ T-cell infiltration or early recurrence rates. However, corticosteroids may influence SRC-1 gene regulation without directly altering cytotoxic T-cell density or immediate tumor progression, and SRC-1 downregulation may be linked to delayed recurrence (59). These observations suggest a possible interaction between corticosteroids and SRC-1–related signaling within brain tumors, although the biological mechanisms remain incompletely understood. Moreover, steroid-mediated immune modulation may occur indirectly through tumor-associated macrophages rather than via a direct SRC-1–CD8^+^ T-cell pathway (**Figure 1**).

### Recent therapeutic strategies co-administered with corticosteroids

Vascular endothelial growth factor (VEGF) secreted by glioblastomas plays a central role in tumor angiogenesis and the development of vasogenic edema (60). Disruption of the neurovascular unit, together with elevated VEGF levels, increases vascular permeability and can lead to life-threatening cerebral edema (61). Experimental elevation of brain VEGF results in marked blood–brain barrier breakdown (62). On this basis, VEGF inhibition represents the most biologically and clinically substantiated steroid-sparing strategy currently available in glioblastoma. Following the introduction of bevacizumab, an anti-VEGF monoclonal antibody, multiple clinical studies demonstrated rapid reduction of vasogenic edema, decreased corticosteroid requirements, and improvement in progression-free survival in selected glioblastoma patients (63). Importantly, bevacizumab remains the only agent with consistent clinical evidence supporting a true steroid-sparing effect, particularly in patients with refractory edema or steroid intolerance. In a murine glioblastoma model, Pitter et al. demonstrated superior efficacy of anti-VEGF therapy compared with dexamethasone (DEX), attributing this to DEX-induced cell-cycle deceleration via p21 upregulation and consequent radioresistance (14).

Angiotensin-II (Ang-II) has been shown to stimulate VEGF production in vascular smooth muscle cells, suggesting that Ang-II blockade may theoretically reduce edema formation (59). *In vivo* studies demonstrated reduced angiogenesis and VEGF expression in mice treated with angiotensin receptor blockers (ARB)or lacking Ang-II receptors (64). Accordingly, angiotensin-converting enzyme inhibitors and angiotensin receptor blockers have been shown to suppress angiogenesis and tumor growth in several experimental models, including glioblastoma (65). However, clinical evidence supporting a steroid-sparing role of these agents remains limited and indirect. Carpentier et al. retrospectively analyzed 87 newly diagnosed glioblastoma patients and reported lower steroid requirements during RT among patients receiving Ang-II inhibitors, without survival benefit (63). These findings should be interpreted cautiously and considered hypothesis-generating rather than practice-changing.

Beyond VEGF-targeted approaches, several agents have been explored primarily in preclinical settings to mitigate corticosteroid-associated adverse effects or resistance mechanisms rather than to replace steroids in clinical practice. Enhancer of Zeste Homolog 2 (EZH2) functions as a histone lysine methyltransferase involved in glioma cell cycle regulation and therapeutic resistance (66). The EZH2 inhibitor UNC1999 demonstrated synergistic antitumor activity with DEX in experimental brain tumor–initiating cell models (67). These findings remain confined to preclinical systems and do not establish a clinical steroid-sparing role.

Riluzole has also been investigated *in vitro* as a strategy to counteract DEX-associated migration and metabolic effects in glioblastoma cells (68). Combined Riluzole and metformin therapy modulated glucose metabolism and stemness pathways in experimental models, increasing tumor cell death, while DEX attenuated migration-related effects (69). Neither riluzole nor metformin is approved or clinically validated as a steroid-sparing therapy in glioblastoma, and their potential utility remains speculative.

Similarly, mifepristone has been studied primarily as a chemosensitizing agent rather than a direct steroid-sparing drug. In experimental glioma models, combined mifepristone–TMZ therapy reduced tumor proliferation and suppressed MGMT expression (70–72). While these data suggest a role in overcoming corticosteroid-associated resistance mechanisms, they do not support routine clinical substitution or reduction of corticosteroids. Chlorpromazine, a repurposed antipsychotic agent, also has shown the ability to enhance TMZ cytotoxicity in preclinical glioblastoma models by disrupting DNA repair pathways (71–73). Its relevance to corticosteroid reduction remains indirect and experimental, and clinical translation has not yet been established.

**Figure 2:**
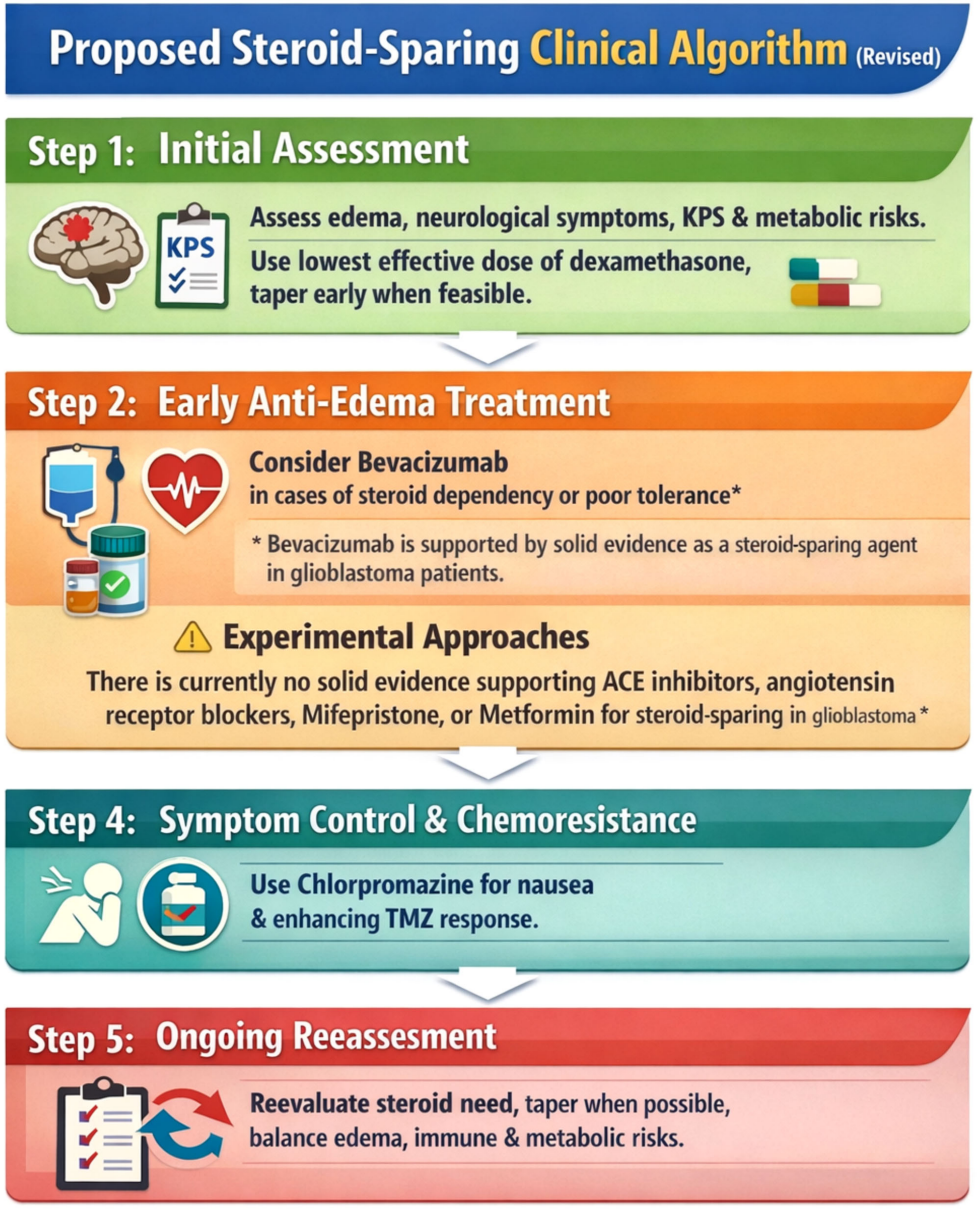
Proposed steroid-sparing clinical algorithm.

A stepwise steroid-sparing clinical algorithm is described in **Figure 2**.

## Conclusion

Corticosteroid exposure in glioblastoma appears to influence tumor progression predominantly through complex immune–microenvironment interactions rather than direct effects on cytotoxic T cells. Steroid molecules reaching the tumor may modulate SRC-1–dependent signaling within glial cells, promoting tumor-supportive pathways that manifest clinically as delayed recurrence. Concurrently, tumor-associated macrophages play a central intermediary role by physically and functionally suppressing CD8^+^ T-cell activity. Steroids may further enhance this macrophage-mediated immune inhibition, either by reinforcing TAM polarization or amplifying their suppressive behavior. Collectively, these mechanisms suggest that the impact of corticosteroids on glioblastoma is driven by SRC-1–linked tumor signaling and TAM-dominated immune regulation rather than direct CD8^+^ T-cell suppression. Emerging strategies, including steroid-sparing approaches and adjunctive therapies that mitigate corticosteroid-related resistance, offer promising avenues. Collectively, these findings emphasize the need for individualized steroid dosing, careful timing relative to oncologic treatments, and continued mechanistic and clinical research to optimize outcomes in glioblastoma patients.
